# Toward Minimal Complexity Models of Membrane Reactors for Hydrogen Production

**DOI:** 10.3390/membranes12111115

**Published:** 2022-11-08

**Authors:** Maria Anna Murmura, Stefano Cerbelli, Ludovica Manozzi, Maria Cristina Annesini

**Affiliations:** Department of Chemical Engineering Materials and Environment, University of Rome “La Sapienza”, Via Eudossiana 18, 00184 Rome, Italy

**Keywords:** Sherwood number, membrane reactor, hydrogen, mass transport, propane dehydrogenation

## Abstract

Membrane reactors are inherently two-dimensional systems that require complex models for an accurate description of the different transport phenomena involved. However, when their performance is limited by mass transport within the reactor rather than by the selective product permeation across the membrane, the 2D model may be significantly simplified. Here we extend results previously found for methane steam reforming membrane reactors to show that such simplified two-dimensional model admits either a straightforward analytical solution for the cross-section averaged concentration profile, or can be reduced to a 1D model with an enhanced Sherwood number, depending on the stoichiometry of the reaction considered. Interestingly, the stoichiometry does not affect the expression of the enhanced Sherwood number, indicating that a versatile tool has been developed for the determination of membrane reactor performance at an extremely low computational cost and good degree of accuracy.

## 1. Introduction

Membrane reactors represent an interesting solution for the decentralized production of high-purity hydrogen, as they allow the integrated production and separation of hydrogen in a single device. In addition, the removal of hydrogen as it is being produced allows to shift the equilibrium of the reaction toward the products, thereby increasing the overall conversion and allowing to carry out endothermic reactions at temperatures lower than those traditionally employed. This solution represents an advantage in terms of energy efficiency, but could also help avoid undesired reactions that take place at higher temperatures, such as coke formation in the presence of hydrocarbons. The use of these devices has been proposed and investigated for many applications, including the reforming of light hydrocarbons [1,2,3], the water-gas shift reaction [4,5], ammonia decomposition [6,7,8], and the dehydrogenation of alkanes [9,10,11]. The simplest reactor configuration envisaged is a tube-in-tube reactor, in which the reaction takes place in the annular volume filled with catalyst particles between the two concentric tubes. Heat may be provided through the outer wall of the tube. A hydrogen-permeable membrane is placed on the outer wall of the inner-most tube. Hydrogen is removed through the selective membrane and flows into the inner tube. A sweep gas is often employed in the permeate side to reduce the partial pressure of hydrogen and increase the driving force for permeation.

In the past years a significant evolution in modeling membrane reactors has been observed, from simple one-dimensional models [12,13] to more complex two- or three-dimensional models [14,15]. While the former models are relatively simple to develop and can be solved at a low computational cost, they could be inaccurate even in predicting integral quantities [16,17]. In addition one-dimensional models seem to be conceptually inadequate for the description of membrane reactors, in which the transport of hydrogen takes place in the direction transversal to that of the main gas flow. Multi-dimensional models, on the other hand, allow to simultaneously account for several transport phenomena and could potentially be very accurate; however, the numerous parameters included in the more complex models are generally difficult to determine with accuracy and the effect of changes in the operating conditions or geometry are more difficult to grasp intuitively from a small set of results, because a single parameter could affect more than one phenomenon.

In this context, the development of one-dimensional models in which transversal concentration gradients are accounted for through a mass transfer coefficient is of interest, as it could combine the benefits of both one- and multi-dimensional models. The difficulty lies in the derivation of an expression for a mass transfer coefficient capable of accounting for the effects not only of convection and dispersion, but also of the reaction and of hydrogen permeation across the selective membrane. Previous experience on modelling membrane reactors for methane steam reforming had shown that, when the resistance to hydrogen permeation across the membrane is negligible, the behavior of the system may be described by a simplified 2D model [18,19]. Here we show that, for reactions characterized by simple stoichiometries, a straightforward analytical solution to the problem exists; whereas for more complex stoichiometries, it is necessary to introduce a mass transfer coefficient. Interestingly, it is found that the stoichiometry of the reaction considered does not affect the expression of the mass transfer coefficient. The present article is divided as follows. In Section 2 the problem is stated in greater detail, the modelling approaches are described in Section 3, and the results are presented in Section 4.

## 2. Statement of the Problem

Previous work on the description of membrane reactors for the production of hydrogen through the steam reforming of methane had highlighted that, depending on the operating conditions, the behavior of such systems could be limited by either radial mass transport within the packed bed or hydrogen permeation across the selective membrane. At a given inlet velocity, the transition between the two regimes was found to depend on the operating pressure. This is due to the fact that while the rate of the convective transport increases almost proportionally with pressure, the rate of permeation depends on the square root of pressure. In fact, hydrogen-selective membranes are generally Pd-based and the rate of permeation depends on the membrane permeability and the difference between the square roots of the hydrogen partial pressures in the retentate and permeate sides according to Sieverts’ law [20]

(1) $$J_{h}=P_{m}\left(\sqrt{p_{H_{2}}^{r}}-\sqrt{p_{H_{2}}^{p}}\right)$$

where $J_{h}$ is the molar hydrogen flux across the membrane, $P_{m}$ is the membrane permeability, $p_{H_{2}}$ is the hydrogen partial pressure, and the superscripts *r* and *p* indicate the retentate and permeate sides, respectively. Under conditions in which the resistance offered by the membrane is negligible, the behavior of the reactor may be described through a simplified two-dimensional model. A more detailed explanation of these observations may be found in [19].

In [21], an expression for an enhanced Sherwood number for the evaluation of the hydrogen concentration profiles in steam reforming membrane reactors was developed, to further simplify the description of the problem to a one-dimensional model in which concentration gradients are accounted for through a mass transfer coefficient, or Sherwood number when the problem is analyzed in its dimensionless form. It is well known that the Sherwood number is a dimensionless group commonly employed to evaluate the mass transport coefficient, $k_{y}$, between the bulk of a fluid, *b*, and a given surface, *s*

(2) $$Sh=\frac{-{\left.\frac{\partial y_{i}}{\partial r}\right|}_{surface}}{y_{i,s}-y_{i,b}}=\frac{k_{y}l_{c}}{D_{i}}$$

and that it expresses the ratio between the rate of mass transport by convection and by diffusion (or dispersion). The Sherwood number is generally evaluated as a function of the Reynolds and Schmidt numbers; however, in reactive systems, the chemical reactions alter the local composition of the mixtures, thereby affecting the species concentration profile and hence the overall transport coefficient. In membrane reactors, there is the additional effect of the selective membrane. Consequently, correlations traditionally employed for the evaluation of the mass transfer coefficient lose significance.

The aim of the present work has been to verify the possibility of extending the findings established in the conetext of steam reforming of hydrocarbons to different reactive systems, specifically to the dehydrogenation of propane. The range of operating conditions in which it is possible to describe the behavior of membrane reactors through this simplified approach with a good degree of accuracy was also investigated. In addition, the possibility of obtaining a simple analytical expression for the simplified 2D model was investigated for reactions characterized by a simple stoichiometry.

The problem was tackled through the following procedure

1. A simplified 2D model, derived in [19], was applied to the propane dehydrogenation reaction;
2. an analytical solution to the average hydrogen concentration profile along the length of the reactor was obtained;
3. an analytical expression for the enhanced Sherwood number was derived and employed in a 1D model of the membrane reactor;

The model was developed for a tube-in-tube catalytic reactor configuration, in which the propane dehydrogenation reaction takes place

(3) $$C_{3}H_{8}\to H_{2}+C_{3}H_{6}\;\;\;\;\;\Delta H^{0}=124.3\;\mathrm{kJ}/\mathrm{mol}$$

Given its endothermicity, this reaction requires high temperatures and is prone to coke formation. To reduce both the energy cost and the risk of catalyst deactivation, it would be useful to lower the operating temperature; on the other hand, this would cause a reduction in the equilibrium conversion of propane. The problem could be solved by employing membrane reactors, in which hydrogen is selectively removed from the reacting volume as it is being produced, thereby shifting the equilibrium of the reaction.

## 3. Modelling

The 2D simplified model was developed under the following simplifying assumptions:

– constant temperature;
– negligible axial dispersion;
– negligible radial convection;
– vanishing hydrogen pressure in the permeate side;
– negligible resistance to hydrogen transport across the membrane;
– constant density;
– local equilibrium conditions

With these assumptions, the mass balance equations read

(4) $$-U\frac{\partial y_{i}}{\partial z}+D_{rr}\left(\frac{1}{r}\frac{\partial y_{i}}{\partial r}+\frac{\partial^{2}y_{i}}{\partial r^{2}}\right)+\nu_{i}r_{p}=0$$

where $y_{i}$ is the molar fraction of the *i*-th component; *U* is the axial velocity, which is constant following the assumption of constant density; $D_{rr}$ is the effective dispersion coefficient in the radial direction, considered to be the same for all components; $\nu_{i}$ is the stoichiometric coefficient of the *i*-th component; $r_{p}$ is the volumetric rate of propane consumption; and the radial and axial coordinates are represented by *r* and *z*, respectively.

The rate of propane dehydrogenation was expressed according to the correlation proposed by Sheintuch et al. [22] while neglecting the inhibiting effect on kinetics by propylene adsorption on the catalyst

(5) $$r_{p}=k_{p}p_{C_{3}H_{8}}\left(1-\eta \right)$$

where $k_{p}$ is the kinetic constant, which, for a given catalyst, depends on temperature only; $p_{C_{3}H_{8}}$ is the partial pressure of propane, whereas the term $\left(1-\eta \right)$ accounts for the distance from chemical equilibrium conditions, with values ranging between 0 and 1

(6) $$\eta =\frac{p_{H_{2}}p_{C_{3}H_{6}}}{p_{C_{3}H_{8}}}\frac{1}{K_{eq}}$$

The boundary conditions are of fixed inlet composition, namely the equilibrium composition under the temperature and pressure conditions considered

(7) $$y_{i}=y_{i}^{0}\;\;\;\;;\;\;\;\mathrm{in}\;z=0$$

impermeability to the flux of all components on the outer wall

(8) $$\frac{\partial y_{i}}{\partial r}=0\;\;\;;\;\;\;\mathrm{in}\;r=R_{2}$$

impermeability of the inner wall to propane and propylene

(9) $$\frac{\partial y_{i}}{\partial r}=0\;\;\;;\;\;\;\mathrm{in}\;r=R_{1}\;\mathrm{for}\;i\neq H_{2}$$

and hydrogen permeation according to Sieverts’ law through the membrane placed on the outer wall of the inner tube

(10) $$\frac{P}{RT}D_{rr}\frac{\partial y_{H_{2}}}{\partial r}=P_{m}\sqrt{p_{H_{2}}}\;\;\;;\;\;\;\mathrm{in}\;r=R_{1}$$

In its dimensionless formulation, Equation (4) becomes

(11) $$-\frac{\partial y_{i}}{\partial \tilde{z}}+\frac{{\tilde{D}}_{rr}}{Pe}\left(\frac{1}{\tilde{r}}\frac{\partial y_{i}}{\partial \tilde{r}}+\frac{\partial^{2}y_{i}}{\partial {\tilde{r}}^{2}}\right)+\nu_{i}Da\;y_{C_{3}H_{8}}\left(1-\eta \right)=0$$

where ${\tilde{D}}_{rr}$ is the dimensionless ratio between the effective dispersion coefficient in the radial direction and the molecular diffusion coefficient, $D_{m}$, the Peclet number

(12) $$Pe=\frac{Ul_{c}}{D_{m}}$$

represents the ratio between the characteristic times of diffusion and convection, and the Damkholer number

(13) $$Da=\frac{RTkl_{c}}{U}$$

The dimensionless axial and radial coordinates were both defined on the basis of the characteristic length, $l_{c}$, defined as the difference between the outer and inner reactor radii

(14) $$l_{c}=R_{2}-R_{1}$$

The boundary conditions remain virtually unchanged, with the exception of the one for hydrogen on the membrane wall, which becomes

(15) $$\frac{{\tilde{D}}_{rr}}{Pe}\frac{\partial y_{i}}{\partial r}=\gamma \tilde{P}\sqrt{y_{H_{2}}}$$

in which a dimensionless parameter representing the ratio between the characteristic times of convection and permeation, γ, is introduced

(16) $$\gamma =\frac{P_{m}RTP_{atm}^{1/2}}{U}$$

having made the operating pressure dimensionless by defining it in terms of the atmospheric pressure

(17) $$\tilde{P}=\frac{P}{P_{atm}}$$

For thin annular channels it is possible to describe the problem in terms of Cartesian coordinates

(18) $$-\frac{\partial y_{i}}{\partial \tilde{z}}+\frac{{\tilde{D}}_{rr}}{Pe}\frac{\partial^{2}y_{i}}{\partial {\tilde{x}}^{2}}+\nu_{i}Day_{C_{3}H_{8}}\left(1-\eta \right)=0$$

From here on, the dimensionless axial and transversal coordinates will be written as *z* and *x*, respectively, for easier readability.

As described in greater detail elsewhere [23] the problem described above may be simplified by considering an auxiliary variable, defined as a linear combination of the molar fractions of hydrogen and of the species from which it is produced, propane in the present case

(19) $$Y=\nu_{H_{2}}y_{C_{3}H_{8}}-\nu_{C_{3}H_{8}}y_{H_{2}}$$

for which the following balance equation holds

(20) $$\frac{\partial Y}{\partial z}-\varepsilon \frac{\partial^{2}Y}{\partial x^{2}}=0$$

with

(21) $$\varepsilon =\frac{{\tilde{D}}_{rr}}{Pe}$$

and

(22) $$x=\frac{r-R_{1}}{R_{2}-R_{1}}$$

To be solved with the boundary conditions

(23) $${\left.Y\right|}_{z=0}=Y^{0}=\nu_{H_{2}}y_{C_{3}H_{8}}^{0}-\nu_{C_{3}H_{8}}y_{H_{2}}^{0}$$

(24) $${\left.\frac{\partial Y}{\partial x}\right|}_{x=1}=0$$

(25) $${\left.Y\right|}_{x=0}=0$$

The latter boundary condition is a consequence of having considered negligible resistance to the transport of hydrogen across the membrane, from which one can set a $y_{H_{2}}=0$ on the membrane wall, and local chemical equilibrium, a condition that can be met only if the partial pressures of hydrogen and propane both go to zero when approaching the membrane wall. The above homogeneous problem admits the following analytical solution

(26) $$Y=2Y^{0}\sum_{l=0}^{\infty }\frac{1}{\lambda_{l}}\exp\left(-\lambda_{l}^{2}\varepsilon z\right)\sin\left(\lambda_{l}x\right)$$

where $\lambda_{l}=\frac{\pi }{2}\left(2l+1\right)$.

Keeping in mind that, given the impermeability of the membrane to all components other than hydrogen,

(27) $${\left.\frac{\partial Y}{\partial x}\right|}_{x=0}={\left.\frac{\partial y_{H_{2}}}{\partial x}\right|}_{x=0}$$

the Sherwood number, defined in Equation (2), is given by

(28) $$Sh\left(z\right)=-2Y^{0}\frac{\sum_{l=0}^{\infty }\exp\left(-\lambda_{l}^{2}\varepsilon z\right)}{{\bar{y}}_{H_{2}}\left(z\right)}$$

where

(29) $$Y^{0}=\nu_{H_{2}}y_{C_{3}H_{8}}^{0}-\nu_{C_{3}H_{8}}y_{H_{2}}^{0}$$

and where ${\bar{y}}_{H_{2}}\left(z\right)$ is the average molar fraction of hydrogen and may be determined by solving the following set of equations

(30a) $$\bar{Y}\left(z\right)=-2Y^{0}\sum_{l=0}^{\infty }\frac{1}{\lambda_{l}^{2}}exp\left(-\lambda_{l}^{2}\varepsilon z\right)$$

(30b) $$\bar{Y}\left(z\right)=\nu_{H_{2}}{\bar{y}}_{C_{3}H_{8}}\left(z\right)-\nu_{C_{3}H_{8}}{\bar{y}}_{H_{2}}\left(z\right)$$

(30c) $$K_{eq}=P\frac{\left(1-{\bar{y}}_{C_{3}H_{8}}-{\bar{y}}_{H_{2}}\right){\bar{y}}_{H_{2}}}{{\bar{y}}_{C_{3}H_{8}}}$$

Given the simple stoichiometry of the reaction, the above set of equations admits the analytical solution

(31) $${\bar{y}}_{H_{2}}=\frac{K_{eq}\bar{Y}\left(z\right)}{P\left(\nu_{H_{2}}-\bar{Y}\left(z\right)\right)-K_{eq}\nu_{C_{3}H_{8}}}$$

Having obtained the above solution, it is interesting to determine how such an average concentration profile would differ from the one obtained with a 1D model with an enhanced Sherwood number and to compare the expression of Sh(z) obtained from the propane dehydrogenation reaction with the one previously obtained for methane steam reforming.

## 4. Results

**Figure 1:**
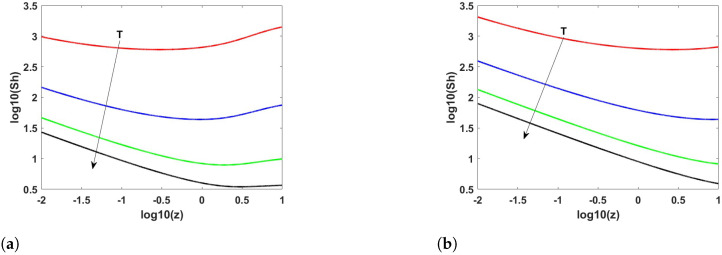
Sherwood number along the length of the reactor at temperatures of 400, 500, 600, and 700 ${}^{{}^{\circ}}$C and 1 atm evaluated from Equation (28) at Pe = 10 (**a**) and 100 (**b**).

Figure 1a,b show the Sherwood number along the length of the reactor, evaluated according to Equation (28) at atmospheric pressure, temperatures between 400 and 700 ${}^{{}^{\circ}}$C and at Pe values of 10 and 100, respectively. With regards to the results shown in Figure 1b, it is interesting to note that at 400 ${}^{{}^{\circ}}$C, the Sherwood number remains almost constant along the entire reactor length. This is mainly attributable to the fact that at low temperatures, the equilibrium conversion of propane is lower than 10%, meaning that the hydrogen concentration is low and the flux permeating across the membrane is not sufficiently high to significantly increase the degree of propane conversion.

The results show that, as in the case of the methane steam reforming reaction, the variation of the Sherwood number along the reactor axis may be described through an expression of the kind

(32) $$Sh\left(z\right)=Sh_{0}\;z^{n}$$

at high values of the Peclet number, whereas the trend for *Sh* changes along the length of the reactor when the Peclet number is low. The different behavior observed depending on the value of Pe was expected because, when all other conditions are kept constant, increasing the value of *Pe* corresponds to a decrease in the dispersion coefficient; consequently, the performance of the reactor is increasingly limited by mass transport within the packed bed, i.e., the main assumption under which the simplified 2D model was developed.

We now move on to consider the same approximated expression for Sh(z) as the one employed for the methane steam reforming reaction, namely [21]

(33) $$Sh\left(z\right)=Sh_{0}{\left(\frac{z}{z^{*}}\right)}^{0.45}$$

with

(34) $$Sh_{0}=-2\Omega^{0}\frac{1}{\nu_{p}}\frac{f_{in}}{W_{h}}\frac{\sum_{l=0}^{\infty }\exp\left(-\lambda_{l}^{2}\varepsilon z\right)}{y_{h}\left(z^{*}\right)}$$

and $z^{*}\ll 1$, and determine the average hydrogen concentration profiles by solving the following problem

(35a) $$\frac{d{\tilde{F}}_{H_{2}}}{d\tilde{z}}=Da\tilde{P}\frac{{\tilde{F}}_{C_{3}H_{8}}}{\sum_{i=1}^{3}{\tilde{F}}_{i}}\left(1-\eta \right)-\frac{Sh}{Pe}\frac{2R_{1}}{R_{2}+R_{1}}\tilde{P}\frac{{\tilde{F}}_{H_{2}}}{\sum_{i=1}^{3}{\tilde{F}}_{i}}$$

(35b) $$\frac{d{\tilde{F}}_{C_{3}H_{8}}}{d\tilde{z}}=-Da\tilde{P}\frac{{\tilde{F}}_{C_{3}H_{8}}}{\sum_{i=1}^{3}{\tilde{F}}_{i}}\left(1-\eta \right)$$

(35c) $$\frac{d{\tilde{F}}_{C_{3}H_{6}}}{d\tilde{z}}=Da\tilde{P}\frac{{\tilde{F}}_{C_{3}H_{8}}}{\sum_{i=1}^{3}{\tilde{F}}_{i}}\left(1-\eta \right)$$

**Figure 2:**
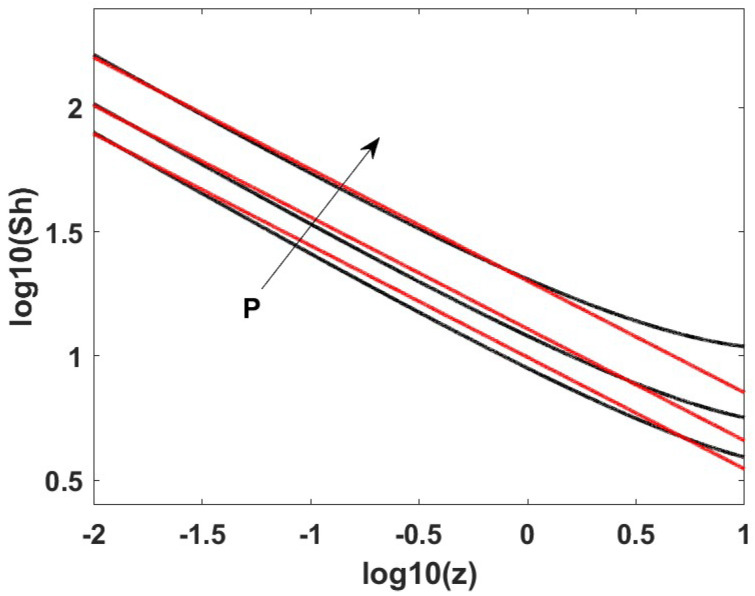
Sherwood number along the length of the reactor evaluated from the simplified 2D model (black curves) and the approximated correlation of Equation (33) (red curves) at Pe = 100, 700 ${}^{{}^{\circ}}$C, and pressures of 1, 3, and 10 bar.

Figure 2 shows a comparison between the values of the Sherwood number along the length of the reactor, evaluated from the simplified 2D model and from Equation (33). The agreement is very good, particularly if taking into account that the approximate expression was simply predicted from results previously obtained for the methane steam reforming reaction.

**Figure 3:**
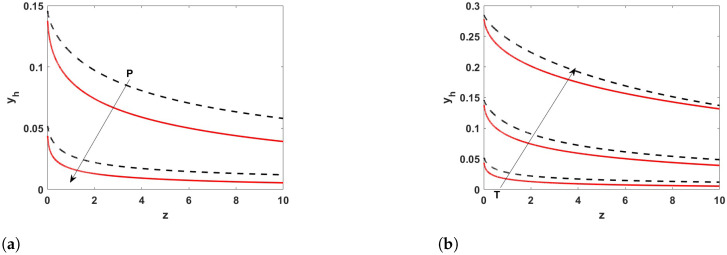
Average hydrogen concentration profiles evaluated from the simplified 2D model (Equation (31), solid red curves) and from the 1D model (Equation (35a–c), dashed black curves) at Pe = 100, and (**a**) T = 500 ${}^{{}^{\circ}}$C at 1 and 10 bar; (**b**) P = 10 bar at 500, 600, and 700 ${}^{{}^{\circ}}$C.

Figure 3 shows the average hydrogen concentration profiles obtained at Pe=100 under different temperature and pressure conditions by solving the 1D model (black dashed curves) of Equations (35a–c) and the simplified 2D model (Equation (31), solid red curves).

**Figure 4:**
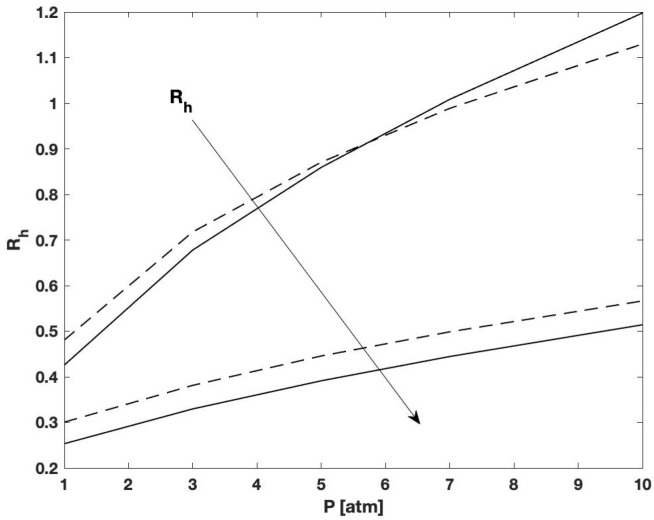
Hydrogen recoveries evaluated from the simplified 2D (solid curves) and 1D (dashed curves) models at Pe = 100, P = 10 atm and temperatures of 600 and 700 ${}^{{}^{\circ}}$C.

It is clear that the agreement between the trends predicted with the two models is excellent, even though the expression for the Sherwood number was not developed specifically for this reactive system. The results also show that there is an error in the evaluation of the hydrogen molar fraction close to the reactor inlet, which causes the discrepancy between the two sets of results. On the other hand, the hydrogen recovery, defined as the ratio between the flowrate of hydrogen permeated across the membrane and the inlet flow rate of hydrogen, evaluated from the two models coincides, as shown from the results reported in Figure 4. This indicates that the proposed solution can be successfully employed for the evaluation of integral quantities at a very low computational cost.

## 5. Conclusions

A simplified 2D model, initially developed to describe the behavior of a membrane reactor for the steam reforming of methane was applied to the propane dehydrogenation reaction in the same type of device. It was shown that, due to the simple stoichiometry of the reaction considered, it is possible to obtain a straightforward analytical expression for the average hydrogen concentration profile along the length of the reactor. Although, in comparison to two-dimensional models, this solution does not allow to have detailed information on the transversal concentration profiles formed within the reactor, significant information is obtained at almost no computational cost. In addition, an attempt was made to describe the performance of the system through a 1D model making use of the same expression for an enhanced Sherwood number previously obtained for the methane steam reforming reactor, with the aim of validating the previously proposed approach. An excellent agreement was obtained between the trends of the average hydrogen concentration profiles along the reactor under conditions in which the behavior of device is limited by mass transport across the reactor bed. The results show that it is possible to evaluate integral quantities with a good degree of accuracy at very low computational cost. The possibility of employing the same expression for Sh(z) regardless of the reactive system indicates that the tool developed is extremely versatile.

## Data Availability

Not applicable.
